# Osseodensification: An Alternative to Conventional Osteotomy in Implant Site Preparation: A Systematic Review

**DOI:** 10.3390/jcm12227046

**Published:** 2023-11-11

**Authors:** João Fontes Pereira, Rosana Costa, Miguel Nunes Vasques, Filomena Salazar, José Manuel Mendes, Marco Infante da Câmara

**Affiliations:** 1 Department of Medicine and Oral Surgery, University Institute of Health Sciences (IUCS-CESPU), 4585-116 Gandra, Portugal; joao.pereira@iucs.cespu.pt (J.F.P.); rosana.costa@iucs.cespu.pt (R.C.); miguel.vasques@iucs.cespu.pt (M.N.V.); filomena.salazar@iucs.cespu.pt (F.S.); 2 Oral Pathology and Rehabilitation Research Unit (UNIPRO), University Institute of Health Sciences (IUCS-CESPU), 4585-116 Gandra, Portugal; jose.mendes@iucs.cespu.pt

**Keywords:** dental implant, osteotomy, osseodensification, sub-antral bone grafts, bone density

## Abstract

Osseodensification is an innovative method of preparing the implant osteotomy using drills that promote bone self-compaction. The main objective of this technique is to promote peri-implant bone densification and compaction of autologous bone and to increase the primary stability of the implant due to the viscoelastic characteristics of the alveolar bone using Densah^®^ burs in a counterclockwise direction at a speed of 800 to 1500 rpm. The objective of this review is the analysis of the scientific literature regarding the applicability of the osseodensification technique in oral implantology. The Preferred Reporting Items for Systematic Reviews and Meta-Analysis guidelines were used and registered at PROSPERO. The search strategy included electronic databases from 2016 to 2023 and was performed by two independent reviewers. The results demonstrate the advantage of the osseodensification technique in relation to conventional drilling, allowing an increase in the bone density and primary stability of the implant, bone density, and bone–implant contact. The osseodensification technique can be applied in different clinical situations: sub-antral bone grafts, narrow alveolar bone crests, low-density bone areas, and immediate implant placement in post-extraction sockets.

## 1. Introduction

The development of the concept of osseointegration by Branemark PI et al. [1] revolutionised the rehabilitation of total and partial edentulous individuals, providing stability and long-term, high success rates in dental implants [1,2,3,4,5]. Osseointegration corresponds to the stable and functional union between the bone and the implant surface, which is crucial for its stability and success [6,7].

Primary stability is considered one of the most important factors for implant success, which is related to the bone density, surgical protocol, type, and geometry of the implant [6,7]. There are methods such as Resonance Frequency Analysis (RFA) or Periotest and insertion torque that can determine implant stability and osseointegration [8,9,10].

In the atrophic posterior maxilla, there is often insufficient residual alveolar bone, which is why it is necessary to increase the base of the maxillary sinus to obtain an adequate volume for the insertion of dental implants. Maxillary sinus elevation was first described by Boyne PV in 1980 [3].

In 1994, Summers R described a technique using a crestal approach using progressive diameter osteotomes that increased the density of the maxillary bone by compaction, allowing the insertion of implants with a high primary stability and the atraumatic elevation of the sinus membrane [4].

Preparation of the implant site can be carried out using the conventional technique of cylindrical or conical drills capable of cutting and extracting bone tissue for the subsequent placement of the implant [11]. However, in 2013, Huwais S introduced an atraumatic osteotomy preparation procedure known as osseodensification (OD) [6]. OD promotes an increase in peri-implant bone density, compaction of autologous bone, plastic deformation of the bone, and increased primary stability of the implant due to the viscoelastic characteristics of the alveolar bone using Densah^®^ drills (2000 Spring Arbor Rd Suite D, Jackson, MI, USA) in a counterclockwise direction at a speed of 800 to 1500 rpm [7]. This technique is indicated in the posterior maxilla in cases of low bone density type IV, sub-antral bone grafts, and in the expansion of narrow bone crests and post-extraction implants [12,13].

The main objective of this systematic review is the analysis of the osseodensification technique as used in sub-antral bone grafts, low-density bone areas, narrow bone crests, and immediate implant placement in post-extraction sockets.

## 2. Materials and Methods

This systematic review was conducted between November 2022 and July 2023 in accordance with the Preferred Reporting Items for Systematic Reviews and Meta-Analysis guidelines (PRISMA) [14], using the MEDLINE database via PubMed, Cochrane Library, Scopus, and Web of Science (from 2013 to 2023) referring to the last 10 years. Studies carried out with humans and animals were included.

The following search strategy used was: (dental implant [MeSH Terms]) AND (osteotomy [MeSH Terms]); ((osteotomy) OR (osseodensification)) AND (dental implants).

The articles were analysed by title, abstract, and full text. The studies included in this review matched all the predefined criteria according to PICOS (“Population”, “Intervention”, “Comparison”, “Results”, “Type of study”) (Table 1). 

The study protocol for this systematic review was registered in the International Prospective of Systematic Reviews (PROSPERO) under the number CRD42023417202.

The eligibility criteria were organised using the PICO method as follows:

The inclusion criteria were articles in English, clinical or experimental studies that compared OD with conventional osteotomy techniques (SD) for the placement of dental implants in humans or animals, and studies that evaluated the performance and safety of OD, such as bone density increase, primary stability, bone–implant contact, success rate, or implant survival. The exclusion criteria were articles with no abstract, studies that did not involve the placement of dental implants, and studies that did not use OD as an intervention.

### 2.1. Extracting Sample Data

The data collected were analysed using a table of results, considering the author, study objective, eligibility criteria, study group and duration, number of implants, osteotomy sequence, anatomical area, and results.

### 2.2. Study Quality and Risk of Bias

To assess the methodological quality of a study and determine the risk of bias in its performance, conduct, or analysis, we used the SYRCLE guidelines for animal studies and the Joanna Briggs Institute (JBI) 2017 guidelines for other studies. For each type of study, a form was filled out using the answers Yes (Y), No (N), Uncertain (UN), and Not Applicable (NA). Two independent examiners (J.F.P/M.I.C) were used to demonstrate intra- and inter-examiner reliability, and the Kappa coefficient test applied in this study resulted in almost perfect agreement (0.81–0.99). The degree of quality of the studies on the relational index used and the number of positive responses to the questions was mostly high, including five articles [6,13,15,16,17], although we also found five studies with moderate evidence [2,12,18,19,20] and seven of low quality [7,8,20,21,22,23,24,25].

### 2.3. Sample Characteristics for Study Quality

To assess the methodological quality of a study and to determine the extent to which a study addressed the possibility of bias in its design, conduct, and analysis, we used the SYRCLE checklist for animal studies and Joanna Briggs Institute (JBI) guidance 2017 for each type of human studies (Case–Control and randomised controlled trials) (Table 2, Table 3 and Table 4). For each type of study, a different questionnaire was conducted using the answers Yes (Y), No (N), Unclear (UN), and Not Applicable (NA).

## 3. Results

### 3.1. Search Results

A total of 3009 articles were initially identified. After excluding duplicates and reading the title and abstract, the remaining articles were analysed in full.

Finally, 17 articles were included. The characteristics of all the studies are included in Table 5.

**Figure 1:**
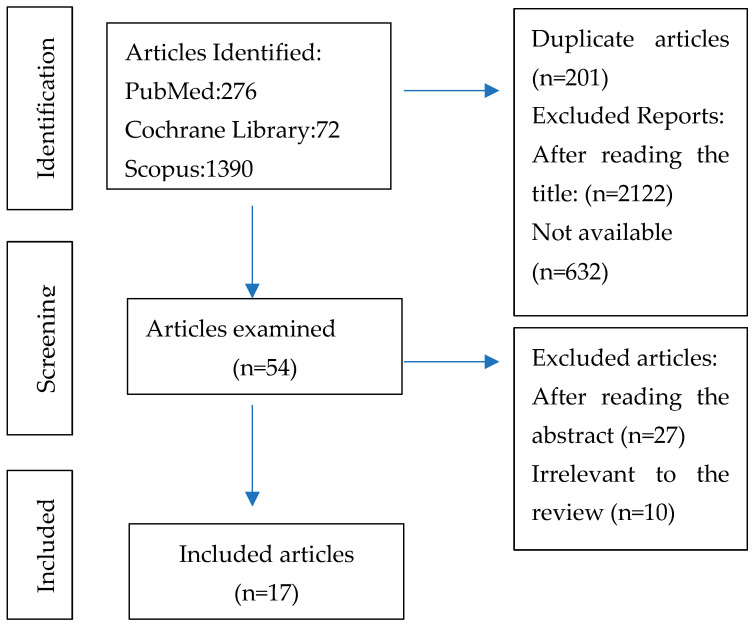
PRISMA flow diagram of study selection.

Figure 1 shows the detailed article selection strategy.

### 3.2. Characteristics of the Included Studies

From each eligible study included in this systematic review, data were collected on general characteristics such as the type of study and objectives, inclusion and exclusion criteria, the study group, and the duration of the study. Data were also collected on the number of implants placed, the anatomical areas where they were placed, and the results obtained (Table 5).

**Table 5:** The main characteristics of the included studies.

Authors	Study Design	Inclusion Criteria	Exclusion Criteria	Study Aim	Study Group	Study Duration	No. Implants	Osteotomy Sequence	Anatomical Zone	Results
Lahens et al. [21], 2016	Experimental study	NR	NR	To investigate the effect of osseodensification on the initial stability and early osseointegration of implants in low-density bone.	Sheep	NR	30	Group SD: Pilot drill 2.0 mm; Twist drill 3.2 mm; Twist drill 3.8 mm. Group OD with *Densah*^®^ *burs:* CW and CCW Pilot drill 2.0 mm; Drill 2.8 mm; Drill 3.8 mm.	Iliac bone	The OD technique showed greater primary stability and greater bone density around the implants compared to the SD technique. Statistical analysis showed that the osseodensification technique promoted a significant increase in the primary stability of the implants (*p* < 0.05). The OD technique showed a higher BIC compared to the SD technique (*p* < 0.05) (±70% and ±50%, respectively). No statistically significant difference in BAFO compared to traditional osteotomy technique (*p* = 0.22); cylindrical implant showed statistically high levels of BAFO compared to conical implants (*p* = 0001).
Trisi et al. [18], 2016	Experimental study	NR	NR	To evaluate a new surgical technique for preparing the implant bed that would improve bone density, ridge width, and secondary implant stability.	Sheep	2 months	20	Group SD: Drilling sequence recommended by the manufacturer. Group OD with *Densah^®^ burs:* Pilot drill 2.0 mm; Drill 2.8 mm; Drill 3.8 mm.	Iliac crest	The OD technique (test group) showed greater primary stability than the SD technique (control group). There was no statistically significant difference in % BIC between the control and test groups (46.19 ± 3.98 vs. 49.58 ± 3.19; *p* > 0.05). Analysis of % BV revealed an increase in bone density of approximately 30 per cent in the test group compared to the control group (37.63 ± 4.25 vs. 28.28 ± 4.74; *p* < 0.05). The test group showed significantly better biomechanical performance (around 30 to 40 per cent higher) than the control group in the parameters assessed, such as RTV (172.70 ± 16.07 vs. 126.63 ± 9.52, *p* < 0.05) and VAM (60.45 ± 5.29 vs. 94.88 ± 10.94, *p* < 0.05).
Huwais and Meyer. [8], 2017	Experimental study	NR	NR	To study the hypothesis that the OD technique would increase primary stability, bone density, and % BIC.	Pigs	NR	72	Group SD: Pilot drill 1.7 mm; Drill 2.2 mm; Drill 3.2 mm; Drill 4.2 mm; Drill 5.2 mm. Group ED: Tapered, multi-fluted bur design. OD group: Pilot drill 1.7 mm; Drill 2.8 mm; Drill 3.8 mm; Drill 4.8 mm; Drill 5.8 mm.	Tibial plateau bone samples	The OD technique showed greater primary stability, bone density, and % BIC compared to the SD and ED techniques. The % BIC was increased by approximately three times for osteotomies prepared with OD compared to SD and ED.
Lopez et al. [22], 2017	Experimental study	NR	NR	To investigate the effectiveness of OD in improving the fixation of spinal surgical material.	Sheep	6 Weeks	36	Group SD (left-sided vertebral body): Pilot drill 2.0 mm; Drill 3.2 mm; Drill 3.8 mm. Group OD (right-sided vertebral body): *Densah^®^ burs* Drill 2.8 mm Drill 3.8 mm	C2, C3, and C4 vertebral bodies	Pullout strength demonstrated that osseodensification drilling provided superior anchoring when compared to the SD group collapsed over time with statistical significance (*p* < 0.01). % BIC analysis demonstrated an OD group with significantly higher values relative to the SD group (*p* < 0.01). % BAFO presented significantly higher values for the OD group compared to the SD group (*p* = 0.024).
Huwais et al. [16], 2018	Multicenter retrospective clinical study	Atrophic partially edentulous posterior maxilla requiring dental implant placement. All patients had crestal sinus augmentation utilising OD and implant placement. Routine: A minimum subsinus vertical bone height of 2 mm. Patients with a minimum of 6 months follow-up from time of augmentation	Sinus pathology that precludes routine sinus augmentation, such as acute sinusitis, history of previous sinus surgery, and bisphosphonate or chronic steroid medications.	To evaluate the effectiveness and predictability of the osseous densification instrumentation method and its ability to facilitate transcrestal sinus elevation with simultaneous implant placement.	115 women,107 men	May 2012 and September 2017	261	*Densah^®^ drills:* Pilot drill 1.7 mm; 3.0 mm drill.	Posterior maxilla	The baseline subsinus residual bone height was 5.4 mm (range: 2–10 mm). Sinus graft augmentation procedure achieved a significant vertical increase of 7 mm (SD: 2.49; *p* < 0.05). No sinus complications were found, such as membrane perforations, and late implant failure was observed in the follow-up period from 6 to 64 months. The cumulative implant survival rate was 97%.
Alifarag et al. [19], 2018	Experimental study	NR	NR	To investigate the effects of OD drilling techniques on implant stability and osseointegration using TM and TSV implants in low-density bone.	Sheep	NR	72:36 TM; 36 TSV.	Group SD: Drill 2.0 mm; Drill 2.8 mm; Drill 3.4 mm. Group OD with *Densah^®^ burs*: Pilot drill 1.7 mm; Drill 2.8 mm; Drill 3.8 mm.	Ilia	TM implants yielded a significantly lower IT (Ncm) relative to the TSV implants (*p* = 0.002). No statistically significant differences across surgical techniques within the TM group despite higher mean values were observed for the OD (CCW and CW) techniques relative to SD. The IT as a function of drilling technique showed implants subjected to SD drilling yielded a significantly lower IT relative to samples implanted in OD (CW/CCW) sites (*p* < 0.05). Histomorphometric analysis showed that OD presented significantly greater values of BIC and BAFO (*p* < 0.05).
Slete et al. [2], 2018	Experimental study	NR	NR	To compare the histomorphometric structure of SD, SO, and a new osteotomy method without bone removal called OD.	Pigs	NR	18	Group SD: Pilot drill 1.7 mm; Manufacturer’s sequence for the appropriate implant size (4.7 mm). Group SO: Pilot drill 1.7 mm; Instrumentation sizes I, II, and III of the set. Group OD with *Densah^®^ burs*: Pilot drill 1.7 mm; Drill 2.5 mm; Drill 3.5 mm; Drill 4.5 mm.	Tibia	OD preparation produced 60.3% of BIC, SO 40.7%, and SD 16.3% of implant perimeter in contact with bone. % BV within 2 mm of implant produced was 62% for OD, 49% for SO. and 54% for SD (compared to SO (40.7%) and SD (16.3%)), with a statistically significant value (*p* < 0.05).
Oliveira et al. [7], 2018	Experimental study	NR	NR	To investigate the effect of OD on the primary stability and osseointegration of machined and acid-etched implants in low-density bone.	Goats	6 Weeks	60	Group SD: Pilot drill 2.0 mm; Drill 3.2 mm; Drill 3.8 mm. Group OD with *Densah^®^ burs* CW and CCW: Pilot drill 2.0 mm; Drill 2.8 mm; Drill 3.8 mm.	Iliac bone	The IT values were approximately 10 Ncm for the SD technique and showed subsequent increases for CW (~53 Ncm) and CCW (~78 Ncm), with statistically significant data as a result of the technique (CCW > CW > SD, *p* < 0.005), regardless of implant surface. % BIC as a function of time (3 vs. 6 weeks); no statistical significance was noted (*p* = 0.577). % BAFO values showed a significant increase in values from 3 to 6 weeks in vivo (*p* = 0.014). Results demonstrated that BIC values for the CCW and CW groups were comparable to all acid-etched implant drilling groups, while the SD drilling for machined groups resulted in significantly lower % BIC values (*p* < 0.01). No significant differences were depicted between acid-etched and machined surfaces when % BAFO values collapsed over time and drilling technique was assessed (*p* = 0.053). Regardless of implant surface, insertion torque significantly increased when OD drilling was used in low-density bone.
Mello-Machado et al. [15], 2018	Case report	NR	NR	To observe whether the clinical and radiographic results obtained could support the hypothesis of gaining primary stability, as well as whether a compaction graft can be achieved using this technique.	Humans	NR	1	*Densah^®^ burs* Pilot drill 1.7 mm; Drill 2.3 mm; Drill 3.0 mm; Drill 3.3 mm.	Maxilla	The OD served to increase primary stability and enhance BIC. The implant was adequately placed and with a sufficient stability, reflected in the ISQ (≥70), which is an indicator of an immediate provisional protocol.
Witek et al. [20], 2019	Case report	NR	NR	To qualitatively and quantitatively evaluate the effect of osteotomy preparation by conventional (control group) or OD (OD group) instrumentation on osteotomy healing.	Sheep	NR	15	Group SD: Pilot drill 2, 3.2, and 3.8 mm twist drills. Group OD *Densah^®^ Burs* OD-CW: Pilot drill 2.0, 2.8 and 3.8 mm multi-fluted tapered burs. OD-CCW: Pilot drill 2.0, 2.8, and 3.8 mm multi-fluted tapered burs.	Left ilium	The mean % BAFO for SD instrumentation was ~11.5%, while both OD techniques (OD-CW and OD-CCW) resulted in statistically homogeneous values: 11.3% and 9.1%, respectively (*p* = 0.78). BAFO values confirmed that there were no healing differences when utilising different instrumentations.
Tian et al. [23], 2019	Experimental study	NR	NR	Comparing the osseointegration of implants placed in atrophic mandibular alveolar ridges with the alveolar ridge expansion surgical protocol.	Pigs	12 + 4 Weeks	12	Conventional osteotomes *Densah^®^ Burs*	Atrophic jaw	The mean % BIC value was approximately 62.5% in the osseodensification group and 31.4% in the regular instrumentation group. Statistical analysis showed a significant effect of the drilling technique (*p* = 0.018). There was no statistical difference in BAFO as a function of drilling technique (*p* = 0.198).
da Rosa et al. [13], 2019	Case report	NR	NR	To describe whether the combined use of IDR and osteotomy through the RE can improve the primary stability of the immediate implant in periodontally compromised extraction sites.	Humans	2 years	2	NR	Maxilla	The combination of the IDR technique with the osseodensification implant site preparation method allowed for an increase in implant primary stability, as demonstrated by the higher insertion torque achieved.
Lahens et al. [24], 2019	Experimental study	NR	NR	To investigate the effects of OD osteotomy on the stability and osseointegration of implants in low-density bone.	Sheep	12 Weeks	72	Group SD: Pilot drill 2.0 mm; Twist drill 3.2 mm; Twist drill 3.8 mm. Group OD *Densah^®^ Burs* (CW and CCW): Pilot drill 2.0 mm; Drill 2.8 mm; Drill 3.8 mm.	Iliac Crest	OD insertion torque was higher in the CCW and CW drilling compared to the SD (*p* < 0.001). BIC was significantly higher for CW (*p* = 0.024) and CCW drilling (*p* = 0.006) compared to the SD technique. BIC values were significantly lower for the SD surgical technique relative to the CCW and CW surgical techniques (*p* < 0.024). The acid-etched surface treatment yielded a significantly higher % BIC than the machine-cut implants (*p* < 0.001). No statistical difference in the BIC as a function of time between the 3-week and 12-week time points (*p* > 0.5). Osseodensification drilling techniques (CW and CCW) yielded significantly higher % BAFO than the SD technique for the acid-etched implants (*p* < 0.01), while in the machine cut implant, the CCW drilling technique yielded a significantly higher BAFO than the SD technique (*p* < 0.01). In low-density bone, OD drilling presented higher stability and no osseointegration impairments compared to the SD technique, regardless of evaluation time or implant surface.
Jarikian et al. [6], 2021	Randomised controlled clinical trial	Good oral hygiene; presence of an edentulous site with an initial width of the alveolar crest between 4 and 5 mm with a minimum of 2 mm of trabecular bone core between the cortical plates.	Uncontrolled systemic conditions or systemic disorders that could compromise osseointegration;consumption of medication that could affect bone metabolism.	To compare the ridge expansion obtained using two different techniques, the OD technique and TET.	Humans	NR	40	TET Group: Pilot drill 1.7 mm; Expander 2.5 mm; Expander 3.1 mm; Expander 3.6 mm. Group OD: *Densah^®^ Burs* Pilot drill 1.7 mm; Drill 2.0 mm; Drill 2.3 mm; Drill 3.3 mm; Drill 3.5 mm.	Alveolar bone	Both techniques were useful in achieving expansion, and all implants placed were successful. The amount of achieved expansion was significantly higher in the OD group, where the average expansion was 2.36 mm (2.36 ± 0.31, *p* < 0.05), while the average amount of expansion in the threaded expanders group was 1.5 mm (1.5 ± 0.28, *p* < 0.05). The Densah bur drilling was superior to manually threaded expanders.
Salgar et al. [17], 2021	Case report	Healthy, non-smoking individuals; requires maxillary sinus augmentation;maximum residual bone height of 1.5 mm.	NR	Presentation of a minimally invasive technique that facilitates bone graft augmentation of the maxillary sinus.	Humans	4 months	5	Group OD: *Densah^®^ Burs* Drill 3.0 mm; Drill 4.0 mm; Drill 5.0 mm; Drill 5.3 mm.	Maxilla	The vertical increase in sinus bone height ranged from 10.3 to 13.6 mm.The rise in bone height is comparable to that obtained with lateral window procedures. The osseodensified crestal sinus window technique may be proposed as a possible alternative procedure for the lateral sinus window technique for maxillary sinus bone augmentation.
Torroni et al. [25], 2021	Case-controlled split model	NR	NR	Comparison of conventional instrumentation vs. OD osteotomy instrumentation in posterior lumbar fixation in an ovine model to determine the feasibility and potential advantages of the OD drilling technique in terms of mechanical and histomorphology outcomes.	Sheep	6 to 12 months	64	Group SD: Pilot drill; Twist drill 4.0 mm. Group OD: *Densah^®^ Burs* Drill 2.8 mm; Drill 3.8 mm.	Lumbar region (spinous processes of L2 to L5)	Considerable mechanical stability differences were observed between OD and SD groups at 6- (387 N vs. 292 N) and 12-week (312 N vs. 212 N) time points. The % BAFO did not yield any significant differences when evaluated as a function of the insertion technique (OD vs. SD (*p* = 0.457)) and time in vivo (*p* = 0.957) The histometric analysis showed no statistical differences in BAFO between SD and OD groups. Mechanical pullout testing demonstrated that OD drilling provided greater degrees of implant anchoring as a function of time, whereas a significant reduction was observed for the SD group.
Mello-Machado et al. [12], 2021	Randomised controlled trial	Patients older than 18 years of age requiring oral rehabilitation of the upper jaw.	Insufficient bone for implant placement; lack of primary stability at implant insertion; metabolic diseases; impeded/ hampered hygiene motor difficulties; pregnancy; uncontrolled periodontal disease.; smoking habits, radio-therapy, and use of bisphosphonates.	To compare the stability of dental implants placed in low-quality bone prepared for the healing chamber with the osseodensification technique and a standard undersized drilling.	Humans	7 months	55	Group SD: Pilot drill 2.0 mm; Drill 2.5 mm; Drill 2.8 mm. Group OD: *Densah^®^ Burs* Pilot drill 1.6 mm; Drill 2.3 mm; Drill 3.0 mm; Drill 3.3 mm.	Upper jaw	The OD group showed higher IT (39.0 ± 6.4 Ncm) than the SD group (32.0 ± 3.4 Ncm) (*p* < 0.001). ISQ values were similar (*p* > 0.05) at the implant insertion (67.1 ± 3.2 and 65.5 ± 2.7, OD vs. SD, respectively). After six months of healing, implant survival was equally comparable in both groups (*p* > 0.05), and ISQ values were greater than those of implant insertion (*p* < 0.001) but similar (*p* > 0.05) for both groups (74.0 ± 3.6 and 73.3 ± 3.2 for OD and SD, respectively) OD instrumentation allowed for the bone-healing chamber concept in low-quality bone without any reduction in implant stability and success rate.

Legend: OD- Osseodensification; BAFO—Bone area fraction occupancy; BIC—Bone-to-implant contact; BV—Bone volume; CCW—Counterclockwise; CW—Clockwise; ED—Extraction drilling; IDR—Immediate dentoalveolar restauration; ISQ—Implant stability quotient; IT—Insertion torque; NR—Non-referred; SO—Summers’ osteotome; RTV—Removal torque value; SD—Conventional osteotomy; TET—Threaded expander surgical technique; TM implants—Trabecular metal implants; TSV implants—Twisted screw-vent implants; VAM- Value of the actual micromotion.

## 4. Discussion

According to the results obtained, the OD technique has advantages over the SD and osteotome techniques in terms of primary implant stability, bone density, BIC, and clinical success of the implants [7,12,15,18,20,21]. The OD technique achieved a greater bone density around implants, greater bone–implant contact, and a higher implant success rate after healing when compared to conventional techniques [2,7,8,18,19,21,24,25]. These results can be explained by the fact that the OD technique preserves and increases the bone matrix during the implant site preparation, which ultimately favours the osseointegration of the implants, as well as allowing additional procedures such as the elevation of the maxillary sinus, the expansion of narrow alveolar ridges, and the prevention of cortical collapse [2,6,13,21,24,26]. These results are in line with the existing literature, which suggests that the OD technique can be a very viable and minimally invasive option for optimising the implant site preparation [17,23,25].

The results obtained in the studies analysed using the technique suggest a better prognosis for dental implants placed in different clinical situations: low-density bone (type IV), narrow alveolar ridges, maxillary sinus grafts, and post-extraction implants [7,8,15].

### 4.1. Insertion Torque and Primary Stability

Several studies have investigated and compared the OD technique and the SD techniques in the context of the primary stability of dental implants. According to Lahens et al. [21], Trisi et al. [18], Huwais and Meyer [8], Alifarag et al. [19], Oliveira et al. [7], Torroni et al. [25], and Mello-Machado et al. [12], OD promotes significantly greater primary stability when compared to SD techniques.

Specifically, when analysing the results related to insertion torque, which is a measure of primary stability, the studies reported that OD had higher insertion torque values compared to SD osteotomy. According to Lahens et al. [21], they observed an average increase of 30% in insertion torque with OD compared to the SD technique, with an average insertion torque value for the SD technique of approximately 10 Ncm and for the OD techniques (CW and CCW) it was significantly higher, with values of over 50 Ncm for CW and around 80 Ncm for CCW. Similarly, Huwais and Meyer [8] reported an average 25% increase in insertion torque with OD.

Alifarag et al. [19] carried out a comparative study and observed an average insertion torque of 45 Ncm with the OD, while the SD technique showed an average insertion torque of only 30 Ncm. In a study by Oliveira et al. [7], similar results were found, with an average insertion torque of 40 Ncm using OD osteotomy and 25 Ncm using the SD technique.

In a study carried out by Trisi et al. [18], statistically significant values of approximately 30% to 40% higher (*p* < 0.05) were observed in relation to primary stability when comparing the OD technique with the SD technique. Mello-Machado et al. [15] obtained an insertion torque of 45 Ncm and an ISQ > 70 when placing the implant using the OD technique, while Mele et al. [26] obtained an ISQ of 74 using the technique in felines.

Oliveira et al. [7], Trisi et al. [18], and Alifarag et al. [19] consistently report that osseodensification is a promising surgical technique that improves the primary stability of dental implants. The osseodensification technique has shown favourable results, measured by insertion torque, indicating greater implant strength and stability in bone tissue compared to conventional osteotomy techniques. These findings highlight the importance and clinical potential of osseodensification in optimising osseointegration [7,18,19].

### 4.2. Bone-to-Implant Contact (BIC) and Bone Area Fraction Occupancy (BAFO)

The osteogenic parameters along the surface of the implants were evaluated by measuring the BIC and the bone growth in the space between the implant spirals as a percentage called BAFO. Animal and human studies have also confirmed that these values tend to increase when using the OD technique.

Tian et al. [23], Trisi et al. [18], Huwais and Meyer [8], Lopez et al. [22], Slete et al. [2], Oliveira et al. [7], Lahens et al. [24], Torroni et al. [25], and Mello-Machado et al. [12] compared the BIC and BAFO values between the OD technique and other SD techniques. The results showed that OD has higher BIC and BAFO values compared to SD osteotomy, although there are variations in the values obtained depending on the implant surface, healing time, and study methodology.

According to Tian et al. [23], OD showed an average BIC value of 80% and BAFO of 70.5%, while with SD osteotomy, the average values were 60% for BIC and 47.5% for BAFO (*p* = 0.018 and *p* = 0.198, respectively). However, according to Torroni et al. [25], there was no significant difference in BIC or BAFO when comparing the different techniques.

Another factor that can influence BIC and BAFO is the type and surface treatment of the implant, as can be seen in the studies carried out by Lahens et al. [21], Alifarag et al. [19], and Oliveira et al. [7]. There are different types of implant designs (parallel, conical), which can be manufactured using different materials (titanium, zirconia, or titanium-zirconia). In addition, there are different implant surface treatments, such as alumina, magnesium oxide, or anodising. According to Oliveira et al. [7], surface treatment with magnesium oxide showed significantly higher BIC and BAFO values than implants with alumina surface treatment in all the osteotomy techniques analysed (*p* < 0.05 BIC and BAFO). The same was found in the study by Lahens et al. [21].

Considering the above, the OD technique improves BIC and BAFO compared to the SD osteotomy techniques.

### 4.3. Osseointegration

Placing implants in the posterior region of the maxilla is a challenge when faced with bone resorption and pneumatisation of the maxillary sinus. To overcome this problem, there are various bone grafting techniques that aim to increase the height and width of the alveolar ridge and prevent the collapse of the buccal cortex. OD is a predictable and advantageous alternative for maxillary sinus elevation and alveolar ridge expansion, improving bone density, primary stability, and osseointegration of dental implants [6,18,24].

The results obtained in the studies suggest that dental implants placed using the OD technique in areas of low bone density or with bone defects have a better prognosis and may reduce the time needed for the implant to achieve osseointegration [7,13,19,21].

OD has emerged as a promising technique in various procedures, especially in clinical situations involving low-density bone. Lahens et al. [21] demonstrated that OD acts as a compacted autotransplant, improving the primary stability of the implant and bone–implant contact. However, further research is needed to better understand the osseointegration process using this technique. Similarly, Lahens et al. [24] highlighted the benefits of OD, indicating that this technique directly influences insertion torque values and improves the stability and osseointegration of endosseous implants in low-density bone, as observed in studies carried out on sheep.

Jarikian et al. [6] emphasised the importance of bone expansion in patients with narrow alveolar ridges, using the OD technique as an effective and less invasive option for increasing the width of the alveolar ridge. Compared to the bone expansion technique with SO, both methods appear to be effective. However, the OD technique was considered more predictable and less invasive. This discussion highlights the importance of proper treatment planning and careful patient assessment to ensure predictable results and minimise complications.

OD has also proved to be a promising technique for maxillary sinus elevation, as described by Salgar et al. [17], whose application of the technique in three patients with difficult clinical situations demonstrated an average increase in bone height of 10.3 mm. OD was able to overcome the limitations of traditional crestal approaches in terms of residual bone height and the limit of vertical height increase, proving to be a minimally invasive option with satisfactory results.

All the results obtained should be analysed and observed with caution since the studies have several limitations and risks of bias, such as the sample size and the short follow-up period. Therefore, more studies with greater methodological rigour and longer follow-up periods are needed to confirm the benefits of the OD technique in oral implantology. In future clinical human trials, it would be worthwhile to perform digitally guided OD in order to evaluate if it improves the promising results of the technique even further [27].

## 5. Conclusions

The studies analysed showed that the OD technique has advantages when used in low-density bone (type IV) by increasing primary stability, bone–implant contact, and clinical success.

In addition, the OD technique can allow for additional procedures such as maxillary sinus elevation, narrow alveolar ridge expansion, and post-extraction implants.

However, these results should be interpreted with caution, as the studies had some limitations and biases. Therefore, more studies with greater methodological rigour and external validity are needed to confirm the benefits of the OD technique in oral implantology.

## Figures and Tables

**Table 1:** PICO’S strategy.

P	Population	Patients who need sub-antral bone grafts or have narrow bone ridges, low-density bone (type IV), and post-extraction implants.
I	Intervention	Use of the OD technique in implant placement.
C	Comparison	Implants placed using other conventional techniques.
O	Outcomes	To analyse the OD technique in oral implantology.

**Table 2:** SYRCLE Checklist for animal studies.

	Was the Attribution Sequence Generated and Applied Properly?	Were the Groups Similar at Baseline, or Were They Adjusted for Confounding Factors in the Analysis?	Has the Distribution of the Different Groups Been Adequately Concealed?	Were the Animals Housed Randomly during the Experiment?	Were the Carers and/or Researchers Blind to the Intervention of Each Animal Received during the Experiment?	Were the Animals Randomly Selected to Evaluate the Results?	Were the Results Assessed or Blind?	Have Incomplete Results Data Been Handled Appropriately?	Are the Study Reports Exempt from Selective Results Reporting?	Was the Study Apparently Free of Other Problems that Could Result in a High Risk of Bias?
Lahens et al. [21], 2016	N	Y	UN	N	N	N	UN	Y	N	N
Trisi et al. [18], 2016	N	Y	UN	N	Y	N	Y	Y	Y	Y
Huwais and Meyer [8], 2017	N	Y	N	UN	N	N	N	N	N	N
Lopez et al. [22], 2017	N	Y	UN	N	N	N	N	N	UN	N
Alifarag et al. [19], 2018	N	Y	N	UN	N	N	N	Y	Y	N
Slete et al. [2], 2018	N	Y	UN	N	N	N	Y	Y	N	N
Oliveira et al. [7], 2018	UN	Y	UN	UN	N	UNr	UN	UN	N	N
Tian et al. [23], 2019	N	Y	UN	UN	N	N	N	Y	UN	UN
Witek et al. [20] 2019	N	Y	N	Y	N	N	N	Y	N	N
Lahens et al. [21], 2019	N	Y	N	UN	N	N	N	Y	N	N
Torroni et al. [25], 2021	N	Y	UN	N	N	N	N	N	N	N

**Table 3:** Joanna Briggs Institute Critical Appraisal Checklist for case reports.

	Have the Demographic Characteristics Been Clearly Described?	Was the Patient’s History Clearly Described and Presented as a Timeline?	Was the Patient’s Current Clinical Condition at the Time of Presentation Clearly Described?	Have the Diagnostic Tests or Methods and the Results Been Clearly Described?	Was the Intervention or Treatment Procedure Clearly Described?	Was the Post-Intervention Clinical State Clearly Described?	Have Adverse Events or Unforeseen Events Been Identified and Described?	Does the Case Report Provide Relevant Data to Draw from?
Mello-Machado et al. [15], 2018	Y	Y	Y	Y	Y	Y	NA	Y
Huwais et al. [16], 2018	N	Y	Y	Y	Y	Y	N	Y
da Rosa et al. [13], 2019	N	N	N	Y	Y	Y	N	Y
Salgar et al. [17], 2021	Y	Y	Y	Y	Y	Y	Y	Y

**Table 4:** Joanna Briggs Institute Critical Appraisal Checklist for randomised controlled clinical trials.

	Was the Randomisation Method Appropriate?	Was the Allocation Method Appropriate?	Were the Groups Similar at the Start of the Study?	Were the Participants Blinded?	Were the Professionals Who Administered the Interventions Blinded?	Were the Outcome Assessors Blinded?	Were the Interventions Clearly Described and Applied Equally to the Groups?	Was the Primary Outcome Clearly Defined and Measured?	Was there an Intention-to-Treat Analysis?	Have Losses and Exclusions Been Described?	Were there any Complications or Adverse Events Reported?	Were the Results of the Study Accurate and Reliable?	Were the Results of the Study Relevant to Clinical Practice?
Jarikian et al. [6], 2021	Y	Y	Y	N	N	Y	Y	Y	Y	N	Y	UN	UN
Mello-Machado et al. [12], 2021	UN	UN	Y	N	N	Y	Y	Y	N	Y	N	UN	UN

## Data Availability

The data can be accessed by contacting the corresponding author.
